# Supplementary material for: Identification of photoperiod-induced specific miRNAs in the adrenal glands of Sunite sheep (Ovis aries)
Source: Front Vet Sci. 2022 Jul 22;9:888207. doi: 10.3389/fvets.2022.888207 (PMC9354845; doi:10.3389/fvets.2022.888207)
Supplement: Supplementary file 1 [file Data_Sheet_1.ZIP › Supplementary file/miRDeep2 file.zip!/miRDeep2 file/Novel_Structure/SP42A2/Novel_277.pdf]

Provisional ID	:	NC_019470.2_26051
Score total	:	1.8
Score for star read(s)	:	-1.3
Score for read counts	:	0
Score for mfe	:	1.5
Score for randfold	:	1.6
Score for cons. seed	:	
Total read count	:	178
Mature read count	:	178
Loop read count	:	0
Star read count	:	0

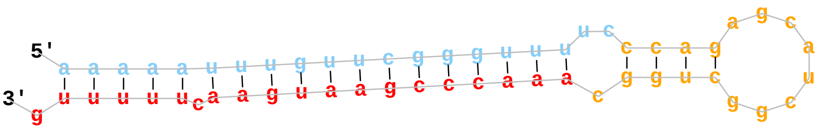

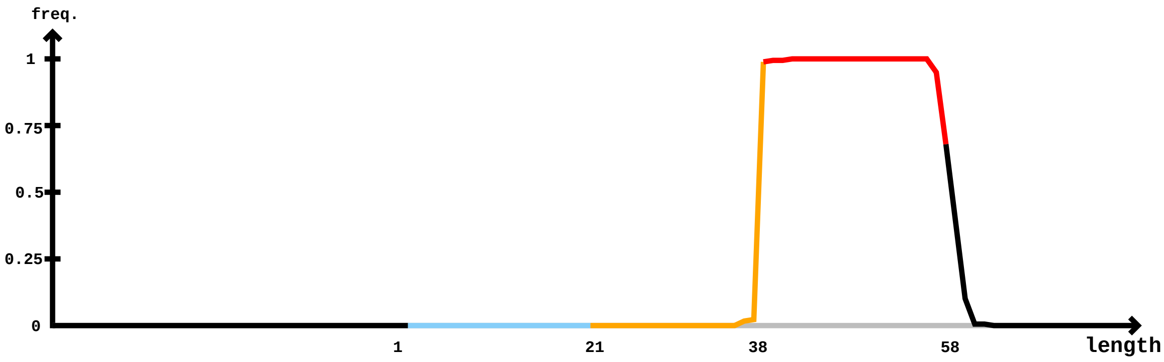

Star Mature

```
5'- guguugaucuaacacacuauaucucagguuggucaaaaaauuuguucggguuuucccagagcaucggcuggcaaacccgaaugaacuuuuuggucaacucacuacucuggc   -3'    exp
    ((((((...))))))...........(((((((((((((.(((((((((((((..((((........)))).))))))))))))).))))))))))))).............   reads      mm         sample
    ......................................................................gcaaacccgaaugaacuuuuu....................   1          0          seq
    ......................................................................gcaaacccgaaugaacuuuuug...................   2          0          seq
    .......................................................................caaacccAaaugaacuuuuu....................   1          1          seq
    ........................................................................aaacccgaaugaacuuuu.....................   9          0          seq
    ........................................................................aaacccgaaugaacuuuuu....................   45         0          seq
    ........................................................................aaacccgaaugaacAuuuu....................   1          1          seq
    ........................................................................aaacccgaaugaacuuuuuA...................   11         1          seq
    ........................................................................aaacccgaaugaacuuuuuC...................   2          1          seq
    ........................................................................aaacccgaaugaacuuuuuU...................   2          1          seq
    ........................................................................aaacccgaaugaacuuuuug...................   85         0          seq
    ........................................................................aaacccgaaugaacuuuuuggu.................   17         0          seq
    .........................................................................aacccgaaugaacuuuuuU...................   1          1          seq
    ...........................................................................Uccgaaugaacuuuuugguca...............   1          1          seq
```
